# The Ethanol-Induced Stimulation of Rat Duodenal Mucosal Bicarbonate Secretion *In Vivo* Is Critically Dependent on Luminal Cl^–^

**DOI:** 10.1371/journal.pone.0102654

**Published:** 2014-07-17

**Authors:** Anna Sommansson, Wan Salman Wan Saudi, Olof Nylander, Markus Sjöblom

**Affiliations:** Division of Gastrointestinal Physiology, Department of Neuroscience, Uppsala University, Uppsala, Sweden; Duke University Medical Center, United States of America

## Abstract

Alcohol may induce metabolic and functional changes in gastrointestinal epithelial cells, contributing to impaired mucosal barrier function. Duodenal mucosal bicarbonate secretion (DBS) is a primary epithelial defense against gastric acid and also has an important function in maintaining the homeostasis of the juxtamucosal microenvironment. The aim in this study was to investigate the effects of the luminal perfusion of moderate concentrations of ethanol *in vivo* on epithelial DBS, fluid secretion and paracellular permeability. Under thiobarbiturate anesthesia, a ∼30-mm segment of the proximal duodenum with an intact blood supply was perfused *in situ* in rats. The effects on DBS, duodenal transepithelial net fluid flux and the blood-to-lumen clearance of ^51^Cr-EDTA were investigated. Perfusing the duodenum with isotonic solutions of 10% or 15% ethanol-by-volume for 30 min increased DBS in a concentration-dependent manner, while the net fluid flux did not change. Pre-treatment with the CFTR inhibitor CFTR_inh172_ (i.p. or i.v.) did not change the secretory response to ethanol, while removing Cl^−^ from the luminal perfusate abolished the ethanol-induced increase in DBS. The administration of hexamethonium (i.v.) but not capsazepine significantly reduced the basal net fluid flux and the ethanol-induced increase in DBS. Perfusing the duodenum with a combination of 1.0 mM HCl and 15% ethanol induced significantly greater increases in DBS than 15% ethanol or 1.0 mM HCl alone but did not influence fluid flux. Our data demonstrate that ethanol induces increases in DBS through a mechanism that is critically dependent on luminal Cl^−^ and partly dependent on enteric neural pathways involving nicotinic receptors. Ethanol and HCl appears to stimulate DBS via the activation of different bicarbonate transporting mechanisms.

## Introduction

Alcoholic beverages are widely consumed throughout the world [1]. Depending on the concentration and the amount ingested, alcohol is considered both a tonic and a toxin.

In the fasting state, approximately 10% of the total ethanol content ingested is absorbed by the gastric epithelium, while the main fraction enters the body via the duodenal mucosa by diffusion [2]. It has long been known that ethanol induces both functional and metabolic changes of the gastrointestinal (GI) epithelium that may result in GI lesions and bleedings. Mucosal damage and ethanol-induced dysmotility lead to the incomplete digestion of nutrients and malnutrition. Ethanol also increases mucosal permeability, allowing endotoxins and other bacterial toxins to more easily enter the body, which contributes to intestinal inflammation.

In experimental animal models, absolute ethanol causes severe damage to the superficial mucosa and focal hemorrhagic lesions extending deep into the mucosa [3]. Chronic exposure to moderate ethanol concentrations is associated with morphological alterations of the upper small intestine including bleb formation and the detachment of the epithelium from the lamina propria [4]. Recent experiments from our laboratory showed that a short duodenal exposure (30 min) of 15% alcohol by volume (ABV) induce low-grade morphological changes in only a small number of duodenal villi tips in rats [5]. Additionally, ethanol induces dysmotility, increases duodenal epithelial paracellular permeability, and stimulates gastric acid secretion as well as pancreatic exocrine secretion [6], [7], [8], [9].

Exposure of ethanol in concentrations higher than 40% is proposed to increase gastric and duodenal bicarbonate secretion (DBS) via increased intercellular leakage [10], [11]. Furthermore, DBS has also been demonstrated to decrease after 15% ABV exposure *in vitro* [12]. The influence of moderately high concentrations of ethanol alone or in combination with acid on DBS *in vivo* has not yet been established.

DBS is an important epithelial defense mechanism against hydrochloric acid that has been discharged from the stomach [13], [14]. The transport of bicarbonate by the duodenal epithelia is primarily an active physiologically regulated mechanism. Bicarbonate transport into the duodenal lumen is mediated via apical Cl^−^/HCO_3_ ¯ exchangers and the cystic fibrosis transmembrane conductance regulator (CFTR) [13], [15], [16], [17], [18]. Different isoforms of the apical anion exchanger Slc26 [Slc26a6 (PAT1), Slc26a3 (DRA) and Slc4a9 (AE4)] are involved in the duodenal Cl^−^/HCO_3_ ¯ exchange and have been immunolocalized at the apical membrane of the intestinal epithelium, predominantly along the villous axis [19], [20], [21], [22]. The CFTR, on the other hand, is primarily expressed in the crypts but is also expressed to some extent in the lower parts of the villi [23]. HCO_3_ ¯ may also reach the lumen via intercellular leakage, although data from rat and mice suggest that this route of transport has little impact on the total luminal alkalinization [13], [24], [25], [26], [27].

The aim of the present study was to investigate the effects of ethanol on the regulation of DBS and transepithelial net fluid flux in overnight fasted rats *in vivo*. To mimic a moderate intake of an ethanol beverage, such as wine or cocktails, the duodenal mucosa was perfused with ethanol solutions of up to 15% ABV alone or in the combination with 1.0 mM HCl.

The secretory effects elicited by ethanol were tested during the pharmacological inhibition of the CFTR or in the absence and presence of luminal Cl^−^ to establish the involvement of the SLC26 solute transporters. We further hypothesized that enteric neural nicotinic receptor-mediated and vanilloid receptor-1-sensitive chemonociceptive pathways are involved in the duodenal mucosal bicarbonate secretory response to ethanol.

## Materials and Methods

### Chemicals and drugs

Hexamethonium chloride (H2138), bovine albumin (A2153), d-gluconic acid sodium salt (G9005), dimethyl sulfoxide (DMSO), capsazepine (C191), mecamylamine hydrochloride (M9020), Tween 80 and the anesthetic 5-ethyl-5-(1′-methyl-propyl)-2-thiobarbiturate (Inactin) were purchased from Sigma-Aldrich (St. Louis, MO, USA). Ethanol 95.5 vol-% (Etax A) was purchased from Solveco Chemicals AB, Täby, Sweden. Parecoxib (Dynastat) was obtained from Apoteket AB, Uppsala, Sweden. 3-[3-trifluoromethyl)phenyl]-5-thiaz olidinylidene]methyl]benzoic acid (CFTR_inh_ 172) was obtained from Tocris Bioscience, Ellisville, MO, USA. ^51^Chromium-labelled ethylenediaminetetraacetate (^51^Cr-EDTA) was purchased from PerkinElmer Life Sciences Inc. (Boston, MA, USA).

### Ethics statement

This study was carried out in strict accordance with the recommendations in the Guide for the Care and Use of Laboratory Animals of the National Institutes of Health. All experiments in the present study were approved by the Uppsala Ethics Committee for Experiments with Animals (Permit Number: C309/10).

### Animals

Male Sprague Dawley rats weighing 210–260 g were obtained from Scanbur AB, Sollentuna, Sweden, or from Taconic, Ejby, Denmark. The animals were maintained under standardized temperature and light conditions (12∶12-h light-dark cycle; temperature, 21–22°C). The rats were kept in cages in groups of two or more and had access to tap water and pelleted food (Type R36; Lantmännen, Kimstad, Sweden) *ad libitum*. The animals were deprived of food (fasted) for 16 hours (overnight) prior to the experiments but had free access to drinking water. The experiments were initiated by intraperitoneally anesthetizing the animal at approximately 8 am with Inactin, 120 mg/kg body weight. To minimize preoperative stress, anesthesia was performed within the Animal Department by the person who had previously handled the animals.

### Surgical procedure

The experiments were performed according to a previously described procedure [5], [9], [28]. In the laboratory, the animals were tracheotomized with a tracheal tube to facilitate respiration, and body temperature was maintained at 37–38°C by a heating pad controlled by a rectal thermistor probe throughout the experiments.

The left femoral artery and the left and right femoral vein were catheterized with PE-50 polyethylene catheters (Becton, Dickinson & Co., Franklin Lakes, NJ, USA). For continuous recordings of the systemic arterial blood pressure, the arterial catheter containing 20 IU/ml heparin isotonic saline was connected to a transducer operating a PowerLab system (AD Instruments, Hastings, UK). The vein was used for drug injections and for the infusion of saline.

A laparotomy was performed, and the common bile duct was catheterized with PE-10 polyethylene tubing close to its entrance into the duodenum (2–3 mm) to prevent pancreatico-biliary juice from entering the duodenum. A piece of soft silicone tubing (Silastic, Dow Corning, Midland, MI, USA 1 mm ID) was introduced into the mouth and gently pushed inside the esophagus, guided through the stomach and pylorus, and secured using ligatures 2–5 mm distal to the pylorus. PE-320 tubing was inserted into the duodenum at approximately 2.5–3.5 cm distal to the pylorus; the tubing was secured using ligatures. The proximal duodenal tubing was connected to a peristaltic pump (Gilson minipuls 3, Villiers, Le Bel, France), and the segment was continuously perfused with a 154 mM sodium chloride solution (saline) at a rate of ∼0.4 ml/min. To complete the surgery, the abdominal cavity was closed with sutures, and the wound was covered with plastic foil. At 30 min after surgery, parecoxib 10 mg·kg^−^^1^ was administered intravenously to reverse the surgery-induced paralysis of the intestine. After surgery, ∼60 min was allowed for cardiovascular, respiratory, and intestinal functions to stabilize before the experiments were commenced.

### Measurement of luminal alkalinization

The rate of luminal alkalinization was determined via back titration of the perfusate to pH 4.90 with 10 mM HCl under continuous gassing (100% N_2_) using pH-stat equipment (Autoburette ABU 901 and pH-stat controller PHM 290, Radiometer, Copenhagen, Denmark). The pH electrode was routinely calibrated with standard buffers before the initiation of the titration. The amount of titrated HCl was considered equivalent to the duodenal mucosal HCO_3_^−^ secretion. The rates of luminal alkalinization are expressed as micromoles of the base secreted per centimeter of the intestine per hour (µmol·cm^−^^1^·h^−^^1^).

### Measurement of fluid flux

The difference in the weight of the collection vials with and without perfusate was used to measure flow over a 10-min interval. The perfusate volumes were determined after correcting for density for each solution. The density of the isotonic saline was arbitrarily set to 1.0. The duodenum was perfused (∼0.4 ml/min) with isotonic saline or other solutions, and the perfusate was collected every 10 min. The net fluid flux across the duodenal mucosa was determined by subtracting the perfusate volume per 10 min from the peristaltic pump volume per 10 min, and the result is expressed as ml fluid per gram of wet tissue weight per hour (ml·g^−^^1^·h^−^^1^). The peristaltic pump volume was determined from the mean of two 10-min samples taken immediately after the termination of each experiment.

### Measurement of duodenal mucosal permeability

After the completion of surgery, ^51^Cr-EDTA was administered i.v. as a bolus of ∼75 µCi followed by a continuous infusion at a rate of ∼50 µCi per hour. The radioactive isotope was diluted in saline and infused at a rate of 1.0 ml⋅hr^−1^. One hour was permitted for tissue equilibration of the ^51^Cr-EDTA. Two blood samples (∼0.3 ml each) were collected during the experiment; the first was collected ten minutes prior to starting the experiment, and the second was collected after ending the experiment. The first blood sample was compensated for via an injection of 0.3 ml 7% bovine albumin solution. After centrifugation, 50 µl of the plasma was removed for radioactivity measurements. The duodenal segment was perfused with saline at a rate of 0.4 ml⋅min^−1^, and the perfusate was collected in 10-min samples. The luminal perfusate and the blood plasma were analyzed for ^51^Cr-activity in a gamma counter (1282 Compugamma CS, Pharmacia, Uppsala, Sweden). A linear equation analysis of the plasma samples was made to obtain a corresponding plasma value for each perfusate sample. The clearance of ^51^Cr-EDTA from the blood to lumen was calculated as described previously [28], and the result is expressed as ml·min^−1^·100 g^−1^.

### Experimental protocol

In all of the experiments, the rate of duodenal bicarbonate secretion (µmol·cm^−^^1^·h^−^^1^), net fluid flux (ml·g^−^^1^·h^−^^1^), systemic arterial blood pressure (mmHg) and body temperature (°C) were monitored continuously and recorded at 10-min intervals. In animals in which the duodenal segment was perfused with a Cl^−^-free solution or hydrochloric acid (pH 3), the mucosal paracellular permeability was assessed by measuring the blood-to-lumen clearance of ^51^Cr-EDTA.

Control experiments were performed by measuring the parameters above with 110-min perfusions of the duodenal segment with isotonic saline (300 mOsm·kg^−^^1^) at a rate of ∼0.4 ml⋅min^−1^.

In the animals luminally exposed to ethanol, the experiments started with the perfusion of the duodenum with saline for 30 min to collect basal data. Thereafter, the duodenum was perfused for 30 min with either a 10% or a 15% ethanol solution made isotonic (300 mOsm·kg^−^^1^) with sodium chloride. The experiment was terminated after another 50-min perfusion with saline.

In animals exposed to a CFTR inhibitor, the experimental protocol was exactly the same as above with the exception that the CFTR inhibitor, i.e., CFTR_inh-172_, was administered either i.v. 2.0 mg·kg^−^^1^or i.p. 2.0 mg·kg^−^^1^ 60 min prior to the start of the experiment.

To test the effects of ethanol during luminal Cl^−^-free conditions, the experiments started with the perfusion of the duodenum for 30 min with saline to collect basal data. Thereafter, the segment was perfused with an isotonic Cl^−^-free solution (150 mM sodium gluconate) for another 30 min followed by the perfusion of a Cl^−^-free 15% ethanol solution for 30 min. The experiment was terminated after another 50-min perfusion with saline.

In the animals pretreated with hexamethonium, the experiments started with the perfusion of the duodenum for 40 min with saline to collect basal data. After that, the nicotinic acetylcholine receptor antagonist hexamethonium was administered i.v. as a bolus dose of 10 mg·kg^−^^1^ followed by a continuous i.v. infusion of 10 mg·kg^−^^1^·h^−^^1^ throughout the experiment. To evaluate the effects of luminal ethanol in animals pretreated with the nicotinic receptor antagonist hexamethonium, the experiment was the same as above for “*Animals exposed to luminal ethanol*”, with the exception that the nicotinic acetylcholine receptor antagonist hexamethonium was administered i.v. as a bolus at a dose of 10 mg·kg^−^^1^10 min before starting the ethanol perfusion followed by a continuous i.v. infusion of 10 mg·kg^−^^1^·h^−^^1^ throughout the experiment.

In the animals pretreated with capsazepine and exposed to ethanol luminally, the duodenum was perfused with saline for 40 min to collect basal data. Thereafter, capsazepine was added to the luminal perfusate to a concentration of 0.25 mM and perfused for another 10 min. The duodenum was then perfused for 30 min with isotonic 15% ethanol solution containing 0.25 mM capsazepine. After ethanol perfusion, the experiment was terminated after another 60-min perfusion with saline.

To mimic the physiological situation, i.e., when ethanol is mixed with gastric acid in the stomach, we tested the effects of luminal ethanol during acidic conditions. The experiments started with the perfusion of the duodenal segment for 30 min with saline to collect basal data. Thereafter, the segment was perfused with an isotonic hydrochloric acid (pH 3, 1.0 mM HCl) solution for another 30 min. The experiment was terminated after another 60-min perfusion with saline. In a second series of experiments, the duodenal segment was first perfused with saline for 30 min and subsequently with a 15% ethanol solution mixed in 1.0 mM (pH 3) hydrochloric acid made isotonic with NaCl for 30 min followed by a 60-min perfusion with saline.

### Histology

In four separate series of experiments, specimens from the duodenal segment were examined histologically. In all four groups, the duodenal segment was first perfused with isotonic saline for 30 min and subsequently perfused with one of the following:

Group I: perfusion with saline for 30 min (n = 4).
Group II: perfusion with 15% ethanol made isotonic with NaCl for 30 min (n = 4).
Group III: perfusion with 1.0 mM hydrochloric acid (pH 3) made isotonic with NaCl for 30 min (n = 4).
Group IV: perfusion with 15% ethanol in 1.0 mM hydrochloric acid (pH 3) made isotonic with NaCl for 30 min (n = 4).

After the experiment, the duodenal segment was immediately fixed in a 10% neutral buffered formalin solution. After fixation, the segment was cut along its length and embedded in paraffin. Sections (4-µm-thick) from the middle part of the segment (∼1.5 cm from the pylorus) were stained with hematoxylin and eosin. All villi in each section were evaluated. The duodenal morphology was assessed via light microscopy by an experienced pathologist who was blinded to the treatment regimes.

### Statistical analysis

The descriptive statistics are expressed as the mean ± SEM with the number of experiments given in parentheses. The statistical significance of data was tested via repeated measures analysis of variance. To test differences within a group, a 1-factor repeated measures ANOVA was used followed by a Tukey post-hoc test. Between groups, a 2-way repeated measures ANOVA was used followed by a Bonferroni post-hoc test. All statistical analyses were performed on an IBM-compatible computer using GraphPad Prism 6.01 software (San Diego, CA, USA). A *P-* value of less than 0.05 was considered significant.

## Results

**Figure 1:**
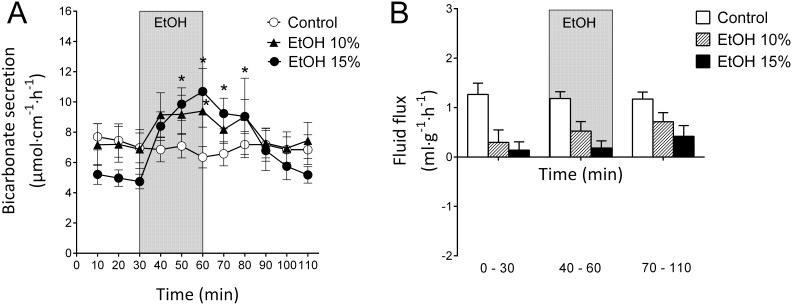
Effects of ethanol on duodenal mucosal bicarbonate secretion and duodenal fluid flux. A). The effects of luminal perfusion of the duodenum with 10% (n = 6) and 15% (n = 11) ethanol on duodenal bicarbonate secretion. Ethanol caused a concentration-dependent increase in duodenal bicarbonate secretion. In the control animals (n = 8, perfusion with isotonic saline only), the bicarbonate secretion was stable during the experiment. B). The luminal perfusion of the duodenum with 10% and 15% ethanol did not have any effects on the duodenal net fluid flux. Similarly, isotonic saline did not influence the net fluid flux in the control animals. However, the basal net fluid flux was significantly (p<0.05) higher in controls compared with that for both ethanol 10% and 15%. The values are the mean ± SEM. *indicates a significant (p<0.05) increase compared with baseline in the same group.

In the controls, in which the duodenal segment was perfused with isotonic saline, bicarbonate secretion (DBS) was stable throughout the entire experiment and averaged 7.00±0.12 µmol cm^−1 ^h^−1^ n = 8 (Fig. 1). The net fluid flux remained fairly stable during the experiment in the same animals. The mean net fluid flux of the 110-min period (1.21±0.27 ml g^−1 ^h^−1^, n = 8) was significantly (p<0.05) different from zero, suggesting net fluid secretion. The mean arterial blood pressure and body temperature remained stable throughout experiments in all of the groups (data not shown).

### Effects of luminal ethanol

Perfusing the duodenal lumen with 10% ethanol during a 30-min interval increased DBS by 36.5% (from 6.88±1.28 to 9.39±1.06 µmol cm^−1 ^h^−1^) (p<0.05, n = 6), as illustrated in Fig. 1A. After the removal of the luminal ethanol, the alkaline secretion returned to basal level. The net fluid flux was 0.30±0.71 ml g^−1 ^h^−1^ during the initial 30-min basal period and did not change during the 10% ethanol exposure (Fig. 1B). Perfusing the duodenal segment with 15% ethanol for 30 min increased the DBS by 126% (from 4.74±0.48 to 10.7±1.53 µmol cm^−1 ^h^−1^) (p<0.05, n = 11, Fig. 1A). After the removal of the luminal ethanol, the bicarbonate secretion returned to basal level. During the same experimental period, the net fluid flux remained unchanged (p>0.05) throughout the experiment; the mean value during the basal period was 0.14±0.53 ml g^−1 ^h^−1^ (Fig. 1B).

### CFTR inhibition and the effects of luminal ethanol

The CFTR inhibitor CFTR_inh-172_ is an inhibitor of CFTR-mediated bicarbonate/chloride transport [29], [30]. In the present study, neither the i.v. nor i.p. pre-administration of the CFTR inhibitor CFTR_inh-172_ altered the duodenal bicarbonate secretory response to luminal ethanol (15%).

**Figure 2:**
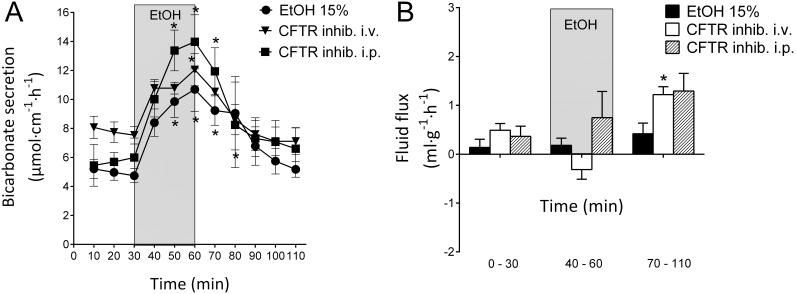
Effects of CFTR inhibition on ethanol-induced increases in duodenal bicarbonate secretion and fluid flux. A). The effects of luminal perfusion of the duodenum with 15% ethanol (n = 11), 15% ethanol pretreated with CFTR_inh-172_ i.v. (n = 6) and 15% ethanol pretreated with CFTR_inh-172_ i.p. (n = 4) on duodenal bicarbonate secretion. Neither the i.v. nor i.p. administration of CFTR_inh-172_ had any effect on the ethanol-induced increase in duodenal bicarbonate secretion. B). Neither the i.v. nor i.p. administration of CFTR_inh-172_ had any effect on the basal or ethanol-stimulated fluid flux. However, in animals administered CFTR_inh-172_ i.v., the net fluid flux increased after ethanol perfusion. The values are the mean ± SEM. * indicates a significant (p<0.05) increase compared with baseline in the same group.

At 60 min prior to the start of the first series of the experiment, CFTR_inh-172_ 2.0 mg·kg^−^^1^ was administered i.v. After the initial 30 min basal period, the duodenal segment was perfused with ethanol at a concentration of 15% for 30 min. The secretory response in this group of animals (from 7.74±0.71 to 12.05±1.10 µmol cm^−1 ^h^−1^, p<0.05, n = 6) was not significantly different from that of the ethanol control group (Fig. 2A). Similarly, with the i.p. pre-administration of CFTR_inh-172_ in the same dose as that for i.v. (2.0 mg·kg^−^^1^) administration, 15% ethanol induced an increase in DBS from 5.70±0.64 to 13.98±1.86 µmol cm^−1 ^h^−1^ (p<0.05, n = 4, Fig. 2A), which did not differ from the increase in the ethanol control group. Additionally, the net output of bicarbonate in response to ethanol exposure did not differ among the ethanol control group, the ethanol + CFTR_inh-172_ i.v. group and the ethanol + CFTR_inh-172_ i.p. group (11.9±2.2, 8.7±1.6 and 15.4±2.6 µmol cm^−1^ 30 min^−1^, respectively).

In the same experiments, the basal net fluid flux was 0.49±0.34 ml g^−1 ^h^−1^ (n = 6) for the i.v. CFTR_inh-172_ pretreatment and 0.36±0.46 ml g^−1^h^−1^ (n = 6) for the i.p. CFTR_inh-172_ pretreatment; these values did not differ from the ethanol control group. Similarly, neither the i.v. nor the i.p. pre-administration of CFTR_inh-172_ influenced the net fluid flux in response to ethanol. These values were not different from those of the controls (p>0.05, Fig. 2B).

### Effects of luminal ethanol during luminal Cl?-free conditions

**Figure 3:**
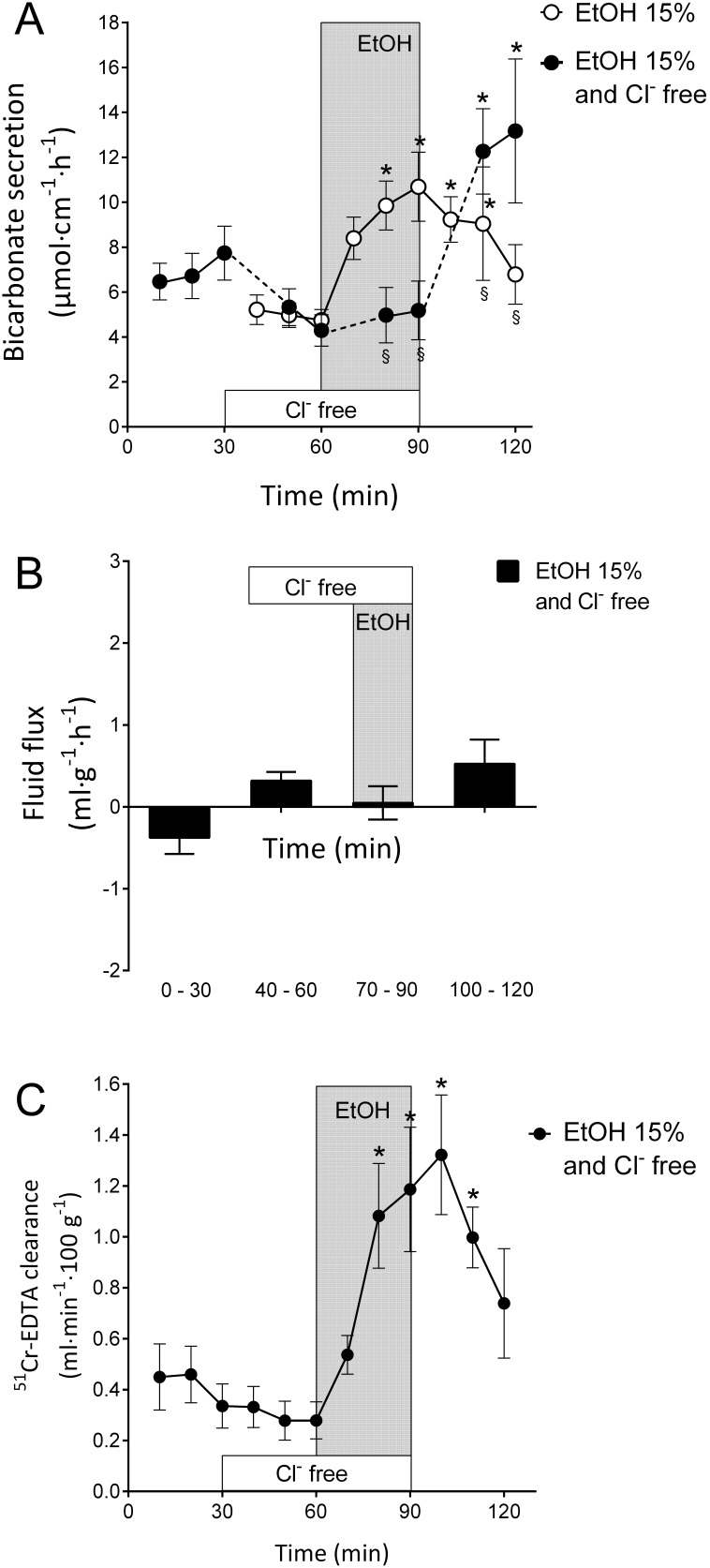
Ethanol-induced stimulation is critically dependent on luminal Cl^−^. A). The effects of luminal perfusion of the duodenum with 15% ethanol during luminal Cl^−^- free conditions on duodenal bicarbonate secretion was investigated. Ethanol did not induce increases in duodenal bicarbonate secretion during Cl^−^-free conditions. B). No statistically significant effects on duodenal net fluid flux were observed in response to the perfusion of ethanol during Cl^−^-free conditions. C). Ethanol precipitated a potent increase in the duodenal-epithelial blood-to-lumen clearance of ^51^Cr-EDTA during Cl^−^-free conditions. The increase in permeability in response to ethanol was in the same magnitude as that observed when luminal Cl^−^ was present (not shown). The values are the mean ± SEM, n = 11 in both groups. * indicates a significant (p<0.05) increase compared with baseline in the same group, and § indicates a significantly lower value compared with the corresponding time point in the other group.

The depletion of Cl^−^ from the duodenal luminal perfusate is an effective experimental approach to abolish ion transport facilitated by apical Cl^−^/HCO_3_? exchangers [15], [31]. Perfusing the duodenal lumen with a Cl^−^-free solution decreased basal bicarbonate secretion from 6.72±1.00 to 4.30±0.70 µmol cm^−1 ^h^−1^ (p<0.05, n = 11, Fig. 3A). Interestingly, perfusing the duodenal segment for 30 min with 15% ethanol in Cl^−^-free conditions did not induce an increase in the rate of bicarbonate transport; the secretion instead remained at a rate of 5.10±0.10 µmol cm^−1 ^h^−1^ (Fig. 3A). The reperfusion of the duodenum with the isotonic NaCl solution induced a potent increase in DBS to 13.16±3.20 µmol cm^−1 ^h^−1^ (p<0.05, n = 11, Fig. 3A). During the same experimental period, the net fluid flux remained unchanged, with a mean value of −0.38±0.60 ml g^−1^h^−1^ (Fig. 3B).

It was recently demonstrated that luminal perfusion with 15% ethanol markedly increased the duodenal clearance of ^51^Cr-EDTA [5]. It was therefore of interest to study the effect of ethanol during Cl–-free conditions. Perfusing the duodenal lumen with 15% ethanol during 30 min in Cl–-free conditions increased the ^51^Cr-EDTA clearance from 0.28±0.07 to 1.32±0.24 ml⋅min^−1^⋅100 g^−1^ (p<0.05, n = 6, Fig. 3C), an increase that was not significantly different from that observed during the administration of 15% ethanol in an isotonic NaCl solution (*c.f.* results previously published in [5] from 0.18±0.06 to 1.67±0.29 ml⋅min^−1^⋅100 g^−1^).

### Effects of luminal ethanol during nicotinic receptor inhibition

**Figure 4:**
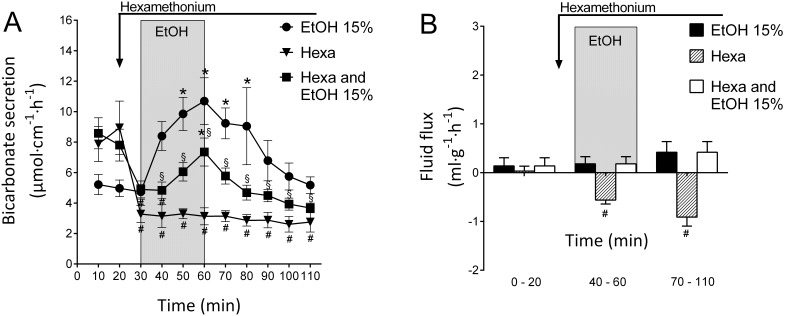
Nicotinic receptor inhibition reduces ethanol-induced increases in duodenal bicarbonate secretion. A). The effects of luminal perfusion of the duodenum with 15% ethanol pretreated with hexamethonium administered as an i.v. bolus dose of 10 mg·kg?^1^ followed by a continuous i.v. infusion of 10 mg·kg?^1^·h?^1^ throughout the experiment. Hexamethonium significantly reduced the bicarbonate secretory response to ethanol. However, during the ethanol exposure in this group, duodenal bicarbonate secretion was significantly increased compared with the preceding basal period. B). The hexamethonium treatment significantly decreased the net fluid flux. In response to luminal ethanol, the net fluid flux was not significantly different from that of the controls. The values are the mean ± SEM; 15% ethanol (n = 11), hexamethonium alone (n = 4), and 15% ethanol + hexamethonium (n = 9). * indicates a significant (p<0.05) increase compared with baseline in the same group, # indicates a significant decrease compared with baseline in the same group and § indicates a significantly lower value compared with the corresponding time point in the ethanol 15% group of animals.

The administration of the nonselective nicotinic receptor antagonist hexamethonium at an i.v. bolus dose of 10 mg·kg^−^^1^ followed by a continuous i.v. infusion of 10 mg·kg^−^^1^·h^−^^1^ throughout experiment decreased the DBS from 7.88±1.16 to 3.13±0.56 µmol cm^−1 ^h^−1^ (p<0.05, n = 4, Fig. 4A). In the present study, hexamethonium caused an instant and significant (p<0.05) drop in the mean arterial blood pressure (from 119±3.4 to 77±5.1 mmHg, not shown). During the same experimental period, the net fluid flux decreased from 0.03±0.20 to a minimum value of −0.91±0.4 ml g^−1^h^−1^ (p<0.05, n = 4, Fig. 4B).

Interestingly, the hexamethonium treatment significantly reduced the increase in DBS induced by 15% ethanol. In hexamethonium-treated rats, the rate of bicarbonate transport increased significantly from 4.90±0.56 to 7.35±0.92 µmol cm^−1 ^h^−1^ in response to perfusion with 15% ethanol (p<0.05, n = 9, Fig. 4A), an increase that was significantly smaller than that induced by ethanol alone. Treatment with hexamethonium also significantly reduced (p<0.05, n = 9) the net output of bicarbonate during the 30 min exposure to 15% ethanol (from 11.9±2.2 to 2.6±0.6 µmol cm^−1^ 30 min^−1^, Fig. 4A). During the same experimental period, the net fluid flux remained unchanged, with a mean value of 0.14±0.53 ml g^−1^h^−1^ (Fig. 4B).

### Effects of luminal ethanol and pretreatment with capsazepine

The ethanol-induced changes in DBS may be partly mediated via the activation of capsaicin receptors, as ethanol was previously demonstrated to potentiate vanilloid receptor 1 function [32]. The luminal administration of the capsaicin antagonist capsazepine (1 µM-1 mM) has been shown to inhibit the acid-induced increase in duodenal blood flow [33] and capsaicin-induced increase in DBS [34]. Recently, it was shown that luminal capsazepine (0.25 mM) inhibited ethanol-induced changes in duodenal motility but did not influence the ethanol-induced increase in mucosal permeability [5].

In the present study, luminal capsazepine (0.25 mM) had no significant (p>0.05) effect on 15% ethanol-induced increases in the DBS and net fluid flux. The increase in DBS in response to ethanol in this group of animals was from 10.9±1.05 to 17.34±1.52 µmol cm^−1 ^h^−1^, p<0.05, n = 9), which was not significantly different (p>0.05) from ethanol alone. Similarly, the net output of bicarbonate in response to ethanol exposure did not differ between the ethanol alone and the ethanol + capsazepine-treated animals (11.9±2.2 and 12.9±2.1 µmol cm^−1^ 30 min^−1^, respectively). In animals treated with luminal capsazepine, the net fluid flux decreased slightly from a basal value of −0.03±0.64 to a value of −1.12±1.22 ml g^−1^h^−1^ during ethanol exposure; however, this change was not significantly different (p>0.05, n = 9) from the control period.

### Effects of luminal ethanol in combination with acid challenge

**Figure 5:**
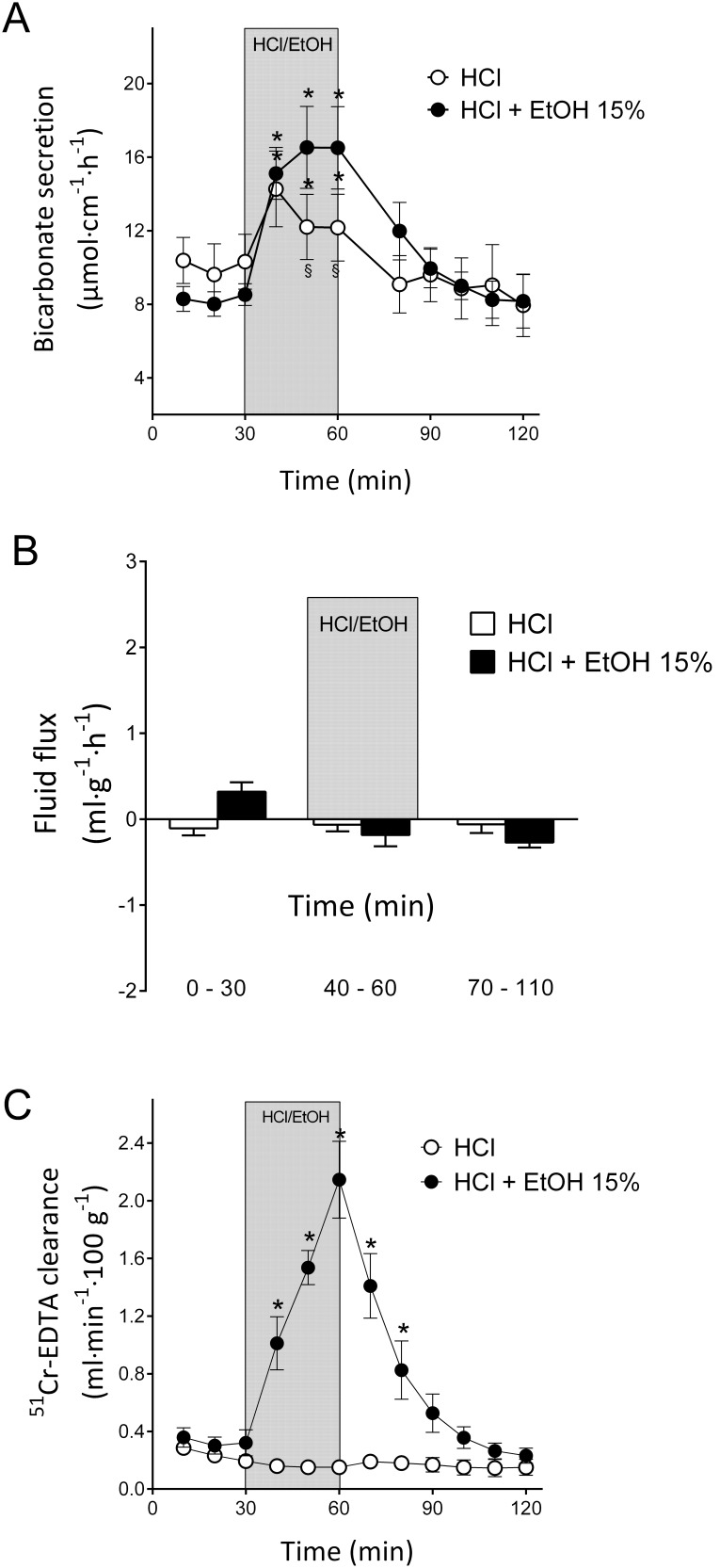
Effects of ethanol and hydrochloric acid on duodenal bicarbonate secretion, paracellular permeability and fluid flux. A). The effects of luminal perfusion of the duodenum with 1.0 mM hydrochloric acid on duodenal bicarbonate secretion were investigated. Hydrochloric acid induced a significant increase in duodenal bicarbonate secretion. A combination of 15% ethanol and 1.0 mM hydrochloric acid induced a significantly larger increase in bicarbonate secretion than hydrochloric acid alone. B). No statistically significant effects on the duodenal net fluid flux were observed in response to hydrochloric acid alone or the combination of 15% ethanol and hydrochloric acid. C). The combination of 15% ethanol and hydrochloric acid caused a potent increase in the duodenal epithelial blood-to-lumen clearance of ^51^Cr-EDTA. However, 1.0 mM hydrochloric acid alone did not influence the clearance of ^51^Cr-EDTA. The values are the mean ± SEM; 1.0 mM hydrochloric acid (pH 3, n = 6), and a combination of 15% ethanol and 1.0 mM hydrochloric acid (n = 7). * indicates a significant (p<0.05) increase compared with baseline in the same group, and § indicates a significantly lower value compared with the corresponding time point in the other group.

The presence of acid in the duodenal lumen was a powerful physiologic stimulus for DBS in all species tested. Interestingly, the changes in DBS and net fluid flux in response to a combination of 15% ethanol and a strong hydrochloric acid solution (i.e., pH<4) have not been elucidated. In the first group of animals tested, the increase in DBS in response to 1.0 mM hydrochloric acid for 30 min was from 10.3±1.49 to 14.3±2.06 µmol cm^−1 ^h^−1^, p<0.05, n = 6, Fig. 5A). The same perfusion did not influence the blood-to-lumen ^51^Cr-EDTA clearance (from 0.19±0.03 to 0.15±0.03 ml⋅min^−1^⋅100 g^−1^ (p>0.05, n = 6, Fig. 5C). Additionally, during the same experimental period, the net fluid flux did not significantly (p>0.05, n = 6, Fig. 5B) change (from a basal value of −0.11±0.20 to a value of −0.063±0.19 ml g^−1^h^−1^) during the hydrochloric acid exposure.

To mimic the condition when ethanol is mixed with gastric acid in the stomach, we perfused the duodenal lumen with 15% ethanol mixed in a 1.0 mM hydrochloric acid solution for 30 min. The DBS increased from 8.52±0.59 to 16.5±2.22 µmol cm^−1 ^h^−1^, p<0.05, n = 6, (Fig. 5A), and the mucosal paracellular permeability (^51^Cr-EDTA clearance) increased from 0.32±0.09 to 2.15±0.27 ml⋅min^−1^⋅100 g^−1^ (p<0.05, n = 6, Fig. 5C); this increase was slightly larger (p<0.05) than that observed during 15% ethanol alone (*c.f.* results previously published in [5] from 0.18±0.06 to 1.67±0.29 ml⋅min^−1^⋅100 g^−1^). Additionally, the net output of bicarbonate in response to 15% ethanol mixed in 1.0 mM hydrochloric acid for 30 min was significantly (p<0.05) higher compared with 1.0 mM hydrochloric acid alone as well as ethanol alone (22.6±4.2, 10.4±2.0 and 11.9±2.2 µmol cm^−1^ 30 min^−1^, respectively). No changes in the net fluid flux (from 0.32±0.29 to −0.19±0.35 ml g^−1^h^−1^) were observed in response to the perfusion of ethanol mixed in hydrochloric acid (n = 6, Fig. 5B).

### Histology

**Figure 6:**
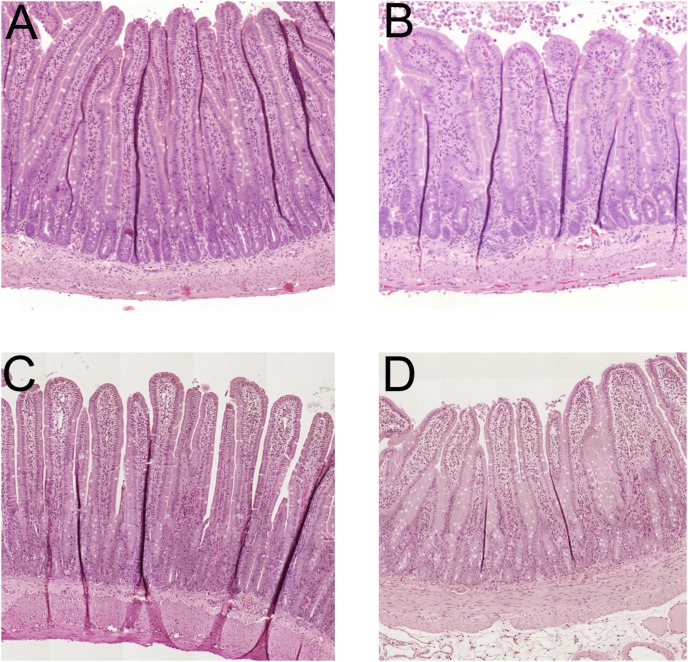
Histology of the duodenal mucosa. The duodenum perfused with isotonic NaCl for 30(group I) had a normal morphological appearance (n = 4, Fig. 6A). The perfusion of the duodenal segment with 15% ethanol for 30 min (group II) caused mild villous tip damage observed as edema and the beginning of desquamation of the epithelium at the tip of less than 10% of the total villi (n = 4, Fig. 6B). The duodenal segment following perfusion of with 1.0 mM hydrochloric acid (pH 3) for 30 min (group III) had a normal morphological appearance (n = 4, Fig. 6C). The perfusion of the duodenal segment with 15% ethanol mixed in a hydrochloric acid solution of 1.0 mM for 30 min (group IV) caused mild villous tip damage observed as edema and the beginning of desquamation of the epithelium at the tip of less than 10% of the total villi (n = 4, Fig. 6D). The morphological changes in this group were not different from the group perfused with 15% ethanol alone.

Figure 6 summarizes the duodenal epithelial morphological appearance after 30 min of perfusion with isotonic saline (n = 4, Fig. 6A), 15% ethanol (n = 4, Fig. 6B), 1.0 mM hydrochloric acid (n = 4, Fig. 6C) and 15% ethanol mixed in a 1.0 mM hydrochloric acid solution (n = 4, Fig. 6D).

## Discussion

The objective of the present study was to determine whether the presence of ethanol in the duodenal lumen induces changes in the rate of DBS and transepithelial net fluid flux. This proved true; we demonstrated for the first time that perfusing the duodenal segment for 30 min with 10% or 15% ethanol in saline induces robust increases in DBS. We further demonstrated that the ethanol-induced secretory increase is critically dependent on the presence of intraluminal Cl^−^, suggesting the activation of apical Cl^−^/HCO_3_? ion exchange. Interestingly, the ethanol-induced increase in bicarbonate secretion is partly mediated via the nervous system because pre-treatment with the nonselective nicotinic receptor antagonist hexamethonium significantly reduced the secretory response. However, perfusing the duodenum with ethanol did not have any effect on the net fluid flux, a finding that is consistent with activation of an electroneutral anion exchange mechanism.

The importance of DBS has been thoroughly established and is currently considered the main epithelial defense mechanism against luminal acid [13], [14]. The rationale for increasing duodenal bicarbonate ion transport in response to ethanol is presently unknown. It could be speculated that ethanol is a general stressor triggering the activation of epithelial defense. In addition, the presence of bicarbonate is important in maintaining a neutral juxtamucosal pH; it provides a stable microclimate that supports the formation of appropriate tight junctional protein complexes [35], [36].

We recently demonstrated that perfusing the duodenal segment with the same concentrations of ethanol as in the present study induces increases in mucosal paracellular permeability [5]. The most rational explanation for the ethanol-induced increase in bicarbonate secretion is epithelial damage, and the increased transport of HCO_3_? would reflect the paracellular passive leakage of bicarbonate from the interstitium to the lumen. The finding that 15% ethanol caused greater increases in DBS and permeability than 10% ethanol supports this notion. However, the histological examinations of the duodenal segment after exposure of 15% ethanol for 30 min revealed minor superficial epithelial changes in only a few villi, and these minor changes were limited to the very tip of the villi [5]. Furthermore, in this study, we found that the bicarbonate secretion returned to baseline level after cessation of the perfusion with the ethanol solution, indicating that the response was reversible. If the ethanol-induced increase in bicarbonate secretion is not primarily caused by mucosal damage, what is the mechanism? In the present investigation, it was demonstrated that perfusing the duodenal lumen with a Cl^−^-free solution decreased basal bicarbonate secretion. This observation confirms previous *in vivo* data and indicates that the absence of Cl^−^ decreases luminal alkalinization by preventing the efflux of HCO_3_? via apical Cl^−^/HCO_3_? exchangers [37]. An interesting finding of the present study was that perfusing the duodenal lumen with a Cl^−^-free ethanol solution prevented an increase in luminal alkalinization. This observation strongly suggests that ethanol activates duodenal epithelial Cl^−^/HCO_3_? exchange. Reintroducing Cl^−^ in the perfusion solution after the ethanol exposure induced a substantial increase in the DBS. The phenomenon of bicarbonate secretion increasing to rates greater than those observed preceding the Cl^−^-free perfusion was previously demonstrated by Pihl *et al* [37]. Although the mechanism behind this increase is not known, it could be speculated that the enterocytes are intracellularly loaded with bicarbonate; bicarbonate cannot exit the apical membrane via Cl^−^/HCO_3_? exchange. When reintroducing chloride at the luminal side, the Cl^−^/HCO_3_ ¯ transporters are activated, and the intracellularly “stored” HCO_3_ ¯ is transported into the lumen at a rate higher than that during basal conditions. Another alternative would be that the luminal Cl^−^-free environment change the setting of the resting secretory state of the CFTR [38], [39]. However, it is beyond the scope of the present study to elucidate the cellular mechanistic machinery by which bicarbonate secretion is increased when reintroducing Cl^−^. However, this is an interesting future objective that warrants further studies. In addition, during the same experimental protocol, no change in net fluid flux was observed, supporting the hypothesis of the activation of an electroneutral anion exchange mechanism. Further supporting the proposal that the bicarbonate secretory response to ethanol involves Cl^−^/HCO_3_? exchange is the finding that neither the intravenous nor intraperitoneal administration of the CFTR inhibitor CFTR_inh-172_ influenced the ethanol-induced increase in luminal alkalinization. Similarly, the administration of CFTR_inh-172_ did not affect the net fluid flux. In the present study, we used the same dose of CFTR_inh-172_ that has previously been demonstrated to inhibit stimulated duodenal bicarbonate secretion but not to have any effect on basal DBS [29]. Additionally, if the ethanol-induced increase in bicarbonate secretion is CFTR dependent, the secretory response would most likely be accompanied by an increase in net fluid flux because CFTR activation also involves fluid secretion [25]. However, in the present study, there was no increase in the net fluid flux in response to luminal ethanol.

To challenge duodenal barrier functions even further and to mimic the physiological situation in which ethanol is mixed with HCl in the stomach, we performed experiments perfusing the duodenal segment with a combination of ethanol (15%) and acid (pH 3) for 30 minutes. It was demonstrated that the duodenal bicarbonate secretory response to the mixed solution was greater than ethanol alone. However, it is important to stress that the net duodenal bicarbonate output in response to ethanol/HCl perfusion was the same net output as the sum of the secretory response to ethanol (15%) and acid (pH 3). The histological examinations of the duodenal segment after the exposure of the mixed ethanol/HCl solution for 30 min did not reveal any epithelial changes that were different from those with ethanol alone. Furthermore, the ethanol/HCl combination induced very similar increases in paracellular permeability, as reported previously for ethanol alone [5]. These observations together suggest that ethanol and HCl stimulates bicarbonate secretion via the activation of different transporting mechanisms in which ethanol is Cl^−^/HCO_3_? exchange dependent and the early secretory phase in response to acid is CFTR dependent [30]. The combination of ethanol and HCl did not have any effect on the net fluid flux.

Mediators released by the nervous system are known to be involved in the regulation of DBS [13], [40]. It has previously been demonstrated that the nicotinic receptor antagonist hexamethonium both decreases DBS and changes the net fluid flux in the absorptive direction. In the present investigation, it was demonstrated that the secretory response was significantly reduced but not abolished when treating the animals with hexamethonium before perfusing the duodenal segment with ethanol. This observation suggests that neural pathways only partly regulate the ethanol-induced increase in DBS. Another interesting observation is that it appears that ethanol inhibits the pro-absorptive effects of hexamethonium, while ethanol alone has no effects on net fluid flux. It could therefore be speculated that the fluid absorption mechanism is disturbed under the influence of ethanol.

We also elucidated whether the vanilloid receptor 1 participates in the mediation of the ethanol-induced increase in DBS. For that purpose, we used the vanilloid receptor 1 antagonist capsazepine at a luminal concentration that was previously demonstrated to inhibit the hyperemic response to luminal CO_2_ in the rat duodenum [33]. Capsazepine did not affect the ethanol-induced bicarbonate secretion or the basal net fluid flux, indicating that the stimulation of vanilloid receptors is not involved in the responses precipitated by ethanol.

The rapid decline in the bicarbonate secretory rate after the removal of the luminal ethanol together with the fact that the increase was abolished during Cl^−^-free conditions and significantly reduced by hexamethonium strongly suggests that the ethanol-stimulated DBS is a regulated mechanism rather than an effect caused by epithelial damage.

In conclusion, exposing the duodenal mucosa to moderately high concentrations of ethanol (similar to those in wine) increases duodenal bicarbonate secretion. We demonstrated for the first time that the secretory response to ethanol occurs via neural pathways involving nicotinic receptors and most likely the activation of the duodenal epithelial Cl^−^/HCO_3_? exchange involving the SLC26 solute transporter family.
